# The roles and controls of GATA factors in blood and cardiac development

**DOI:** 10.1002/iub.2178

**Published:** 2019-11-28

**Authors:** Tomasz Dobrzycki, Mukesh Lalwani, Caroline Telfer, Rui Monteiro, Roger Patient

**Affiliations:** ^1^ Molecular Haematology Unit, Weatherall Institute of Molecular Medicine, John Radcliffe Hospital University of Oxford Oxford UK; ^2^ Institute of Cancer and Genomic Sciences College of Medical and Dental Sciences, IBR West University of Birmingham, Edgbaston Birmingham UK; ^3^ BHF Centre of Research Excellence Oxford UK

**Keywords:** blood, development, GATA factors, gene regulatory networks, heart, regeneration

## Abstract

GATA factors play central roles in the programming of blood and cardiac cells during embryonic development. Using the experimentally accessible Xenopus and zebrafish models, we report observations regarding the roles of GATA‐2 in the development of blood stem cells and GATA‐4, ‐5, and ‐6 in cardiac development. We show that blood stem cells develop from the dorsal lateral plate mesoderm and GATA‐2 is required at multiple stages. Firstly, GATA‐2 is required to make the cells responsive to VEGF‐A signalling by driving the synthesis of its receptor, FLK‐1/KDR. This leads to differentiation into the endothelial cells that form the dorsal aorta. GATA‐2 is again required for the endothelial‐to‐haematopoietic transition that takes place later in the floor of the dorsal aorta. GATA‐2 expression is dependent on BMP signalling for each of these inputs into blood stem cell programming. GATA‐4, ‐5, and ‐6 work together to ensure the specification of cardiac cells during development. We have demonstrated redundancy within the family and also some evolution of the functions of the different family members. Interestingly, one of the features that varies in evolution is the timing of expression relative to other key regulators such as Nkx2.5 and BMP. We show that the GATA factors, Nkx2.5 and BMP regulate each other and it would appear that what is critical is the mutually supportive network of expression rather than the order of expression of each of the component genes. In Xenopus and zebrafish, the cardiac mesoderm is adjacent to an anterior population of cells giving rise to blood and endothelium. This population is not present in mammals and we have shown that, like the cardiac population, the blood and endothelial precursors require GATA‐4, ‐5, and ‐6 for their development. Later, blood‐specific or cardiac‐specific regulators determine the ultimate fate of the cells, and we show that these regulators act cross‐antagonistically. Fibroblast growth factor (FGF) signalling drives the cardiac fate, and we propose that the anterior extension of the FGF signalling field during evolution led to the recruitment of the blood and endothelial precursors into the heart field ultimately resulting in a larger four chambered heart. Zebrafish are able to successfully regenerate their hearts after injury. To understand the pathways involved, with a view to determining why humans cannot do this, we profiled gene expression in the cardiomyocytes before and after injury, and compared those proximal to the injury with those more distal. We were able to identify an enhancement of the expression of regulators of the canonical Wnt pathway proximal to the injury, suggesting that changes in Wnt signalling are responsible for the repair response to injury.

**Abbreviations**
ALPM: anterior lateral plate mesoderm
ATAC‐seq: assay for transposase‐accessible chromatin‐sequencing
BMP: bone morphogenetic protein
CHT: caudal haematopoietic tissue
CM: cardiomyocyte
DA: dorsal aorta
DH: definitive haemangioblast
DLP: dorsal lateral plate
Dpi: days post injury
DPP: decapentaplegic
EGFP: enhanced green fluorescent protein
EHT: endothelial to haematopoietic transition
FACS: fluorescence activated cell sorting
GFP: green fluorescent protein
GO: gene ontology
hpf: hours post fertilisation
hpi: hours post injury
HSC: haematopoietic stem cell
MI: myocardial infarction
MO: morpholino
MY: million years
RNA‐seq: RNA‐sequencing
TGF‐β: transforming growth factor‐β
VEGF‐A: vascular endothelial growth factor‐A

## 1 BLOOD

GATA factors were first discovered in the erythroid cells of the blood, specifically the first three members of the family.^1^ We have been interested in the development of the blood stem cell, in part with a view to generating these cells in vitro for transplantation into humans in the clinic. The GATA factor of particular interest in the formation of blood stem cells is GATA‐2.

Blood or haematopoietic stem cells (HSCs) are found in the bone marrow of mammals, including humans. However, they are made during embryonic development outside the bone marrow and subsequently migrate to it. Therefore to understand how to make HSCs, it is necessary to study their generation in the embryo. This is quite difficult to do for obvious reasons in human embryos. We therefore use the experimentally accessible amphibian (Xenopus) and zebrafish embryos, and the mechanisms discovered there have been shown to be applicable to mammals including humans where tested.^2^, ^3^ Blood is generated multiple times in vertebrate embryos, resulting in the production of predominantly red blood cells early during development to facilitate gaseous exchange. These cells are derived directly from mesoderm and not from a blood stem cell. The stem cell is made later in the dorsal aorta by a process known as the endothelial to haematopoietic transition (EHT).^4^, ^5^, ^6^, ^7^ By labelling cells in the very early embryo and following their fate, we were able to show that the HSC lineage is distinct from the earlier blood lineages.^8^ This was an important discovery because it meant that the HSC lineage would be programmed independently of the earlier blood lineages, receiving signals from distinct surrounding tissues at different times.

Our lineage analysis showed that the HSCs are derived from the most dorsal lateral plate (DLP) mesoderm, which is located immediately adjacent to the forming somites. Programming of these cells involves signals from the somites. The cells then migrate under the somites towards the midline of the embryo where they form a cord of cells that lumenises and forms the dorsal aorta (DA).^9^, ^10^ Under the influence of local signals, the floor of the DA is induced to become haemogenic endothelium, which undergoes EHT and generates HSCs, that are found associated with the floor of the DA.^11^, ^12^ Thus, the dorsal lateral plate mesoderm gives rise to both blood (HSCs) and endothelium and as such we have referred to the cells as haemangioblasts. To distinguish them from the haemangioblasts derived from more ventral mesoderm that gives rise to the primitive blood populations, we have called them definitive haemangioblasts (DHs). Gene expression profiling of definitive and primitive haemangioblasts revealed that while the primitive programme is dominated by blood‐associated genes, the definitive programme is dominated by endothelial associated genes.^13^ This likely reflects the immediate fate of the cells with the definitive cells forming endothelium first and blood (HSCs) only later, while the primitive cells make blood immediately.

## 2 THE GENE REGULATORY NETWORK CONTROLLING DEFINITIVE HAEMANGIOBLAST PROGRAMMING

VEGF‐A is the inducing signal affecting the DLP/DH and its expression in the somites is positively regulated by the ETS factor, ETV‐6.^9^ Interestingly, an early response within the DLP/DHs is the expression of ETV‐6 and therefore VEGF‐A, potentially making the cells independent of the somites. In order to respond to VEGF‐A signalling, the DLP/DHs need to express the VEGF‐A receptor, KDR/FLK‐1. This we have shown is dependent on the ETS transcription factor, FLI‐1, and the GATA factor, GATA‐2. FLI‐1 and GATA‐2 are also required for the expression of the key haematopoietic transcription factor, SCL/TAL‐1, and its partner LMO‐2.^14^ Thus, clearly the control of FLI‐1 and GATA‐2 expression is key to understanding the programming of the mesoderm giving rise to HSCs. Insights into how FLI‐1 is regulated came from work we were doing with micro‐RNAs. We found that FLI‐1 expression is dependent on the presence of miR‐142 that acts by inhibiting the expression of a TGF‐β receptor.^15^ Thus FLI‐1 expression depends on the down regulation of TGF‐β signalling.

In the case of GATA‐2, we speculated that BMP signalling would be involved because both blood development and GATA factor expression have many connections with BMP signalling in the literature. However, BMP is required for the whole of lateral plate mesoderm formation and general patterning of the early embryo. Therefore, to test the specific role around the time when GATA‐2 expression was turned on in the DLP/DHs, we had to make use of conditional inhibition of BMP signalling.^16^ We therefore obtained a transgenic Xenopus line expressing the BMP inhibitor, Noggin, from a heat shock‐inducible promoter, and separately a chemical inhibitor of BMP signalling that could be added to the water housing the experimental embryos. Success of the BMP inhibition in each case was monitored by the presence of phosphorylated SMAD‐1, 5 or 8, the downstream mediators of BMP signalling. We found that BMP input is required at three points during the development of the HSCs: firstly, for the initial expression of GATA‐2 in the DLP/DHs, later for the expression of KDR/FLK‐1 required for the response to VEGF‐A signalling, and thirdly for the expression of GATA‐2 in the DA.^16^

## 3 THE REGULATION OF GATA‐2 EXPRESSION

Evidence published by others^17^ has identified a key role for GATA‐2b in HSC emergence from the DA in zebrafish. Zebrafish underwent a whole genome duplication 100 MY ago and, while some of the duplicates have been lost, many genes still retain duplicated copies. GATA‐2 is one of these. To understand the control of GATA‐2 expression in more detail, we decided to exploit the two copies of GATA‐2 in zebrafish to sub‐functionalise that control.^18^ We searched the two zebrafish GATA‐2 loci for the mouse GATA‐2 enhancer identified by the Engel and Bresnick groups.^19^, ^20^ As well as sequence alignment, we carried out ATAC‐seq on purified endothelial cells isolated by FACS from a KDRL‐GFP transgenic fish line. A correlate of the mouse gene enhancer was found only in GATA‐2a but not in GATA‐2b. The function of this putative enhancer was tested by cloning it upstream of a fluorescent reporter gene, and it was found that all endothelial cells were labelled, thus recapitulating the activity seen in the mouse. We also deleted this putative enhancer from the endogenous GATA‐2a gene and found that endothelial expression was significantly reduced. We therefore conclude that the GATA‐2a enhancer is, as for the mouse GATA‐2 gene, a significant regulator of the endothelial expression of zebrafish GATA‐2a. When we monitored expression of the blood stem cell marker, RUNX‐1, in the fish line expressing a fluorescent reporter under the control of this GATA‐2a enhancer, we found that a subset of the fluorescent cells in the DA were positive for RUNX‐1. This confirmed that GATA‐2a is indeed expressed in the cells destined to become HSCs. By counting cells positive for RUNX‐1 in the mutant line missing the GATA‐2a enhancer, we were able to show that GATA‐2a expression is indeed required for the formation of RUNX‐1 expressing cells in the DA. In other words, the earlier expression of GATA‐2a in the endothelial cells of the DA, as well as the later expression of GATA‐2b in the floor of the DA, is required for the normal formation of HSCs in the DA. To better understand the relationship between GATA‐2a and b, we looked at GATA‐2b expression in the GATA‐2a enhancer mutants. We found that GATA‐2b expression was reduced. Furthermore, GATA‐2b expression driven by the GATA‐2a enhancer could rescue expression of downstream RUNX‐1 expression. Therefore GATA‐2a is upstream of GATA‐2b and RUNX‐1 in the gene regulatory cascade leading to HSC development in the DA.^18^

Despite the significant reduction in RUNX‐1 expressing cells in the DA in the GATA‐2a enhancer mutants at 26 hours post fertilisation (hpf), we found that the number of derivatives of the HSC found at 48 hpf in the caudal haematopoietic tissue (CHT) and the thymus was unaffected.^18^ Furthermore, we found a significant increase in infections and heart oedemas in adult mutant fish compared to wild type, which correlated with reduced cellularity in the kidney marrow, the homologous site to the mammalian bone marrow. This is reminiscent of the human condition caused by GATA‐2 haploinsufficiency^21^ and this line may facilitate the study of this condition.

## 4 GATA‐4, ‐5, AND ‐6 IN CARDIAC DEVELOPMENT AND REGENERATION

### 4.1 Development

GATA‐4, ‐5, and ‐6 are all expressed in the developing myocardium but knockout of individual GATA factors in the mouse has in general failed to abolish myocardial specification,^22^, ^23^, ^24^, ^25^, ^26^ raising the possibility that they may have redundant activities during this process, a concept made more likely by the observation that they all bind to the same GATA‐binding sites with high affinity. To test for redundancy, we set out to knock down each of the genes individually and in combination^27^ using antisense morpholinos (MOs). To avoid individual quirks of either Xenopus or zebrafish, this was carried out in both species. Each of the MOs was validated for specificity in at least one context. To quantitate the effects, we counted the number of embryos in different knock down levels for cardiac marker genes such as myosin light chain, ranging from no reduction of in situ hybridisation signal to complete absence. For both species, knock down of GATA‐4 had very little effect, as did GATA‐5 knock down in Xenopus. However, in both species GATA‐6 knock down had a clear effect that was further enhanced by knocking down GATA‐4 or ‐5 in the same embryos. Likewise, even though knock down of GATA‐4 or ‐5 individually had little effect in Xenopus, a clear phenotype was seen when they were both knocked down. Thus, we have been able to generate clear evidence for redundancy within the cardiac GATA family: a factor such as GATA‐4, which has little effect on its own, can clearly lead to a more severe phenotype when one of the other members of the family is also knocked down.

As mentioned above, knock down of GATA‐5 in Xenopus had little effect on its own.^27^ However, knock down of GATA‐5 on its own in zebrafish had a major effect.^27^, ^28^ This highlights changes in use of the GATA factors during evolution. A possible explanation may derive from the earlier expression of GATA‐5 in zebrafish, reflecting its more important role in specifying cardiac tissue. Interestingly, this means that GATA‐5 is expressed before the critical cardiac specifying gene NKX‐2.5, which is active before GATA factors in Xenopus. These two factors positively regulate each other in both species, suggesting that once either is expressed the other will follow. Apparently, the establishment of this mutually supportive circuit is more important than the order in which the two component genes become active. This concept could be widened further when we looked at the key signalling molecule, bone morphogenetic protein (BMP), which is active before either GATA or NKX factors in zebrafish, whereas NKX is active before BMP in Xenopus. We conclude that the setting up of the mutually supportive circuit is more important than the order in which the component genes become active. Such a circuit is also present in Drosophila, starting with BMP (known as DPP in Drosophila).^27^, ^29^ Davidson and Erwin have called such vital core gene regulatory circuits, kernels.^30^

Cardiac tissue derives from the anterior lateral plate mesoderm (ALPM) where GATA‐4, ‐5, and ‐6 are co‐expressed.^31^ In both Xenopus and zebrafish embryos, the cardiac mesoderm is adjacent to mesoderm giving rise to early blood and endothelial populations. We have therefore referred to this adjacent ALPM as the anterior haemangioblast. Such a population of haemangioblasts is not thought to be present in mammals but rather the whole ALPM contributes to the heart. This made us wonder if the anterior haemangioblast mesoderm had been recruited into the heart field during evolution, giving rise to the larger four chambered heart. Consistent with a close relationship between these two mesodermal populations we showed that GATA‐4, ‐5, and ‐6 are required for the programming of the anterior haemangioblast, as seen for the cardiac mesoderm.^31^ We went on to show that cardiac determinants such as NKX‐2.5 are only expressed in the cardiac ALPM whereas blood determinants such as SCL/TAL‐1 are expressed only in the haemangioblast ALPM.^32^ Furthermore we were able to show that these determinants have a cross‐antagonistic relationship whereby NKX‐2.5 suppresses the blood phenotype and SCL/TAL‐1 suppresses the cardiac phenotype. We also showed that fibroblast growth factor (FGF) favours the cardiac phenotype over the haemangioblast phenotype. FGF patterns the embryo along the antero‐posterior axis and we therefore proposed that this evolutionary recruitment of anterior haemangioblast mesoderm into the cardiac field could have been achieved by extending the anterior boundary of FGF expression.

### 4.2 Regeneration

A common cause of heart failure in humans is the inability of cardiomyocytes (CMs) to proliferate and regenerate the lost myocardium after myocardial infarction (MI). We therefore carried out RNA sequencing (RNA‐seq) analysis of injury‐reactivated CMs in the zebrafish, where regeneration of the heart occurs, to identify spatially distinct gene expression profiles within injured ventricles. Importantly, we found that canonical Wnt antagonists were enriched in proliferating CMs proximal to the injury zone, suggesting a potential role in governing CM proliferation during heart regeneration.

To study this, the Tg(*gata4*:EGFP) zebrafish model was employed.^33^ This transgenic (Tg) line expresses EGFP in a subpopulation of CMs after cardiac injury and lineage tracing showed that these cells proliferate to regenerate the injured area.^33^ We quantified *gata4*:EGFP+ CMs following injury using FACS analysis at 5 hours postinjury (hpi) and 4 days postinjury (dpi). A dramatic increase in the number of *gata4*:EGFP+ cells in the injury‐proximal region was observed, whereas the number was unchanged in the distal region. This observation strongly suggests that injury‐proximal *gata4*:EGFP+ CM cells expand by proliferation and indicates spatio‐temporal heterogeneity within CMs after injury. To identify molecular changes underlying reactivation of CM proliferation following injury, we performed an RNA‐seq experiment on injury‐activated *gata4*:EGFP+ CMs at 4dpi. To understand the selective induction of proliferation in proximal *gata4*:EGFP+ CMs, we carried out two‐way comparisons of RNA‐seq transcriptome data. Firstly, the transcriptome of FACS sorted *gata4*:EGFP+ CMs from whole ventricles at 4 dpi was compared to that of *myl7*:EGFP+ CMs from whole ventricles of sham‐operated animals. This identified 967 genes that are upregulated in the *gata4*:EGFP+ CMs. Secondly, ventricles of 4dpi Tg(*gata4*:EGFP) fish were cut into two, to separate the injury‐proximal and ‐distal regions before FACS isolation of *gata4*:EGFP+ CMs for spatial comparison within the injured heart. This analysis identified 956 genes upregulated in the injury‐proximal region. By intersecting these data sets, we identified 808 genes specifically upregulated in the injury‐proximal signature of *gata4*:EGFP+ CMs, 820 genes in the distal signature and 149 injury‐activated genes that are shared between both regions.

Comparing gene expression profiles of the injury‐proximal and ‐distal regions suggested injury induced, spatially resolved molecular responses. Gene ontology (GO)‐term analysis indicated that CMs in the injured zone were enriched for cell division, angiogenesis, wound healing, and amino acid metabolism, consistent with their active role in proliferation and regenerating myocardium. In contrast, CMs in the distal myocardium were enriched for genes associated with heart and skeletal development, cell differentiation, and organ morphogenesis. The GO‐terms enriched within the proximal data set were also characterised by regulators of growth such as Wnt signalling. Interestingly, we found that positive and negative regulators of canonical Wnt signalling were enriched in the proximal signature,^34^, ^35^, ^36^ and known target genes of Wnt signalling were differentially expressed in CMs from the injury‐proximal region. We therefore hypothesize that, following injury, the homeostasis of Wnt signalling is required to attain a progenitor‐like state and subsequently proliferation. Recently, Wu et al developed tomo‐seq, a method providing spatially resolved genome‐wide expression profiles in whole heart ventricle of zebrafish following cryoinjury, and found that proximal to the injury area there are two regions (injury and border zone) with distinct gene expression signatures in the cryoinjured heart.^37^ We have compared our expression data with the tomo‐seq study and found that some Wnt antagonists were enriched in both zones, whereas others were enriched in the border zone relative to injury.^37^ This comparison strongly suggests cell‐to‐cell variation in the injury response and molecular differences underlying cellular heterogeneity in the CM subpopulation within the injured ventricle. Overall, our RNA‐seq data suggest that canonical Wnt signalling might be reactivated in CMs following injury and required for the proliferative response.
